# Natural History of Echocardiographic and Hemodynamic Changes Following Isolated Pericardiectomy for Constrictive Pericarditis

**DOI:** 10.1186/1749-8090-10-S1-A63

**Published:** 2015-12-16

**Authors:** Erin A Gillaspie, John M Stulak, Richard C Daly, Kevin G Greason, Lyle D Joyce, Jae K Oh, Hartzell V Schaff, Joseph A Dearani

**Affiliations:** 1 Division of Cardiovascular Surgery, Mayo Clinic College of Medicine, Rochester, MN, 55905, USA; 2 Division of Cardiovascular Diseases, Mayo Clinic College of Medicine, Rochester, MN, 55905, USA

## Background/Introduction

Pericardiectomy has been shown to improve the functional status of patients with constrictive pericarditis and resultant heart failure symptoms. However, there are few studies following the chronic hemodynamic impact of surgery on the left ventricle, right ventricle and tricuspid valve.

## Aims/Objectives

We sought to identify a homogenous cohort of patients undergoing surgery for constrictive pericarditis. Echocardiographic data would be collected through the length of their followup, assessing for left ventricular function, right ventricular function and valvular dysfunction to help to better understand the long term outcomes of pericardiectomy.

## Method

January 1993 to December 2013, 938 patients underwent pericardiectomy at our institution. To establish a homogeneous population, we included patients with constrictive pericarditis and excluded patients with prior chest radiation and concomitant valvular or coronary procedures.

## Results

We identified a cohort of 355 patients. Median age at operation was 62 (range 18-84) and 282 (79%) were male. Median pre-operative NYHA Functional class was III and 300/356 (84%) patients were in class III/IV. All patients underwent isolated pericardiectomy; early mortality was 2.5%. During median follow-up of 29 months (max 20.5 yr), 507 echocardiograms were reviewed for tricuspid regurgitation (TR), right ventricular (RV) dysfunction, RV systolic pressure (RVSP), and left ventricular ejection fraction (LVEF). TR grade increased during follow-up from trivial to mild (p = 0.02). Despite this finding, there was no demonstrable impact on RV function or RVSP. Additionally, LVEF remained stable over follow-up. Median NYHA Functional class at last follow-up was I (77% class I/II).

## Discussion/Conclusion

Pericardiectomy is safe and provides significant improvement in functional status during late follow-up. A concern with surgery is the potential for ventricular dilation and dysfunction post-operatively. Our data demonstrate patients undergoing pericardiectomy for constriction have stable ventricular function in late follow-up. There is an observed increase in TR grade from trivial to mild, but we did not observe an associated functional or hemodynamic consequence.

